# Lack of evidence that nephrolithiasis increases the risk of sialolithiasis: A longitudinal follow-up study using a national sample cohort

**DOI:** 10.1371/journal.pone.0196659

**Published:** 2018-04-26

**Authors:** Hyo Geun Choi, Woojin Bang, Bumjung Park, Songyong Sim, Kyung Tae, Chang Myeon Song

**Affiliations:** 1 Department of Otolaryngology-Head and Neck Surgery, Hallym University College of Medicine, Anyang, Korea; 2 Department of Urology, Hallym University Sacred Heart Hospital, Anyang, Korea; 3 Department of Statistics, Hallym University, Chuncheon, Korea; 4 Department of Otolaryngology-Head and Neck Surgery, Hanyang University College of Medicine, Seoul, Korea; University of California San Diego, UNITED STATES

## Abstract

**Objective:**

The objective of this study was to evaluate the risk of sialolithiasis in nephrolithiasis patients.

**Methods:**

Using data from the national cohort study from the Korean Health Insurance Review and Assessment Service, we selected 24,038 patients with nephrolithiasis. The control group consisted of 96,152 participants without nephrolithiasis who were matched 1:4 by age, sex, income, region of residence, diabetes, hypertension, and dyslipidemia. The incidence of sialolithiasis in the two groups was compared, with a follow-up period of up to 12 years. The crude and adjusted hazard ratio (HR) of nephrolithiasis to sialolithiasis was analyzed with a Cox-proportional hazard regression model.

**Results:**

The rates of sialolithiasis in the nephrolithiasis group and the control group were not significantly different (0.08% vs. 0.1%, P = 0.447). The crude and adjusted hazard ratios of nephrolithiasis to sialolithiasis were not statistically significant (crude HR = 0.82, 95% confidence interval [CI] = 0.50–1.35, P = 0.448; adjusted HR = 0.81, 95% CI = 0.49–1.33, P = 0.399). Subgroup analyses according to age and sex also failed to reveal statistical significance.

**Conclusion:**

There is no evidence of an increased risk of sialolithiasis associated with nephrolithiasis. We suggest that routine evaluation for sialolithiasis in all patients with nephrolithiasis is not necessary.

## Introduction

Nephrolithiasis, defined as one or more stones in the kidney, affects more than 6% of the general population [1]. The incidence and prevalence of nephrolithiasis has increased consistently during the last 50 years [2, 3]. The annual incidence rates of nephrolithiasis in the literature are between 85 and 350 per 100,000 [4]. The prevalence rate including renal stones found by ultrasonography in asymptomatic random healthy subjects was 2.0% in two studies [5, 6]. Changes in dietary intake and climate may account for this increase [7, 8].

The exact cause of renal stone formation remains unclear. Various risk factors have been proposed, including chronic kidney disease, poor hydration, increasing age, obesity, diabetes mellitus, warm climate, and high animal protein intake [2, 4, 9]. Abnormalities in calcium metabolism may also be a risk factor for nephrolithiasis, because about 80% of renal stones are composed of calcium substrates. Renal stones are generally classified into five types according to their origin and mineral composition: calcium stones, struvite or magnesium ammonium phosphate stones, uric acid stones, cysteine stones, and drug-induced stones [10]. The most common constituents of renal stones are calcium oxalate (56–61%), calcium phosphate (8–18%), and uric acid (9–17%) [2].

Sialolithiasis is a common stone disorder of the salivary gland or duct that causes swelling or pain in the face or neck by obstructing the discharge of saliva [11]. The annual incidence of sialolithiasis in the recent literature ranges from 28 to 141 per million [12–14]. However, its prevalence including asymptomatic salivary stones is higher; it was reported to be up to 1.2 per 100 people in an autopsy study [15]. In another postmortem study the incidence of microcalculi in the submandibular gland was 80% on microscopic examination [16].

Patients with nephrolithiasis are not routinely examined for stones in other organs. However, because salivary stones are mainly composed of calcium carbonates and calcium phosphates, [12] some investigators have suggested that there may be an association between sialolithiasis and nephrolithiasis, based on the idea of a common pathophysiology [17,18]. In contrast, several retrospective case-control studies and case series found no correlation between sialolithiasis and nephrolithiasis [19–21].

This study aimed to evaluate whether nephrolithiasis is a risk factor for sialolithiasis using a large, nation-wide population-based cohort. study. A nephrolithiasis and control group were matched 1:4 for age, group, sex, region of residence, income group, and past medical histories. To identify the causal relationship between nephrolithiasis and sialolithiasis, patients with sialolithiasis diagnosed before nephrolithiasis were excluded from the study [22].

## Materials and methods

### Study population and data collection

The ethics committee of Hallym University (2014-I148) approved this study. Written informed consent was exempted by the Institutional Review Board.

This national cohort study relies on data from the Korean Health Insurance Review and Assessment Service—National Sample Cohort (HIRA-NSC). The Korean National Health Insurance Service (NHIS) selects samples directly from the entire population database to prevent non-sampling errors. Approximately 2% (one million) of the total number of individuals were selected from the Korean population (50 million). This selected data can be classified into 1,476 levels (age [18 categories], sex [2 categories], and income level [41 categories]) using randomized stratified systematic sampling methods via proportional allocation to represent the entire population. After data selection, the appropriateness of the sample was verified by a statistician who compared the data from the entire Korean population with the sample data. The details of the methods used to perform these procedures are provided by the National Health Insurance Sharing Service [23]. This cohort database included (i) personal information, (ii) health insurance claim codes (procedures and prescriptions), (iii) diagnostic codes using the International Classification of Disease-10 (ICD-10), (iv) death records from the Korean National Statistical Office (using the Korean Standard Classification of disease), (v) socio-economic data (residence and income), and (vi) medical examination data for each participant from 2002 to 2013.

Because all Korean citizens are recognized by a 13-digit resident registration number from birth to death, population statistics can be determined using this database. All Koreans must enroll in the NHIS, and all Korean hospitals and clinics use the 13-digit resident registration number to register individual patients in the medical insurance system. Therefore, the risk of overlapping medical records is minimal, even if a patient moves from one place to another. Moreover, all medical treatments in Korea can be tracked without exception using the HIRA system.

### Participant selection

We identified all participants diagnosed with nephrolithiasis (ICD-10: N20; Calculus of kidney and ureter) among the 1,125,691 individuals with 114,369,638 medical claim codes and selected those who were treated ≥ 2 times (n = 24,107). These participants were followed-up for up to 12 years. Sialolithiasis was diagnosed according to the ICD-10 codes (K115), and we again selected those who were treated ≥ 2 times. From 2002 through 2013, 973 sialolithiasis cases were selected. Cases involving a single visit to a clinic were excluded to reduce false positives.

**Fig 1:**
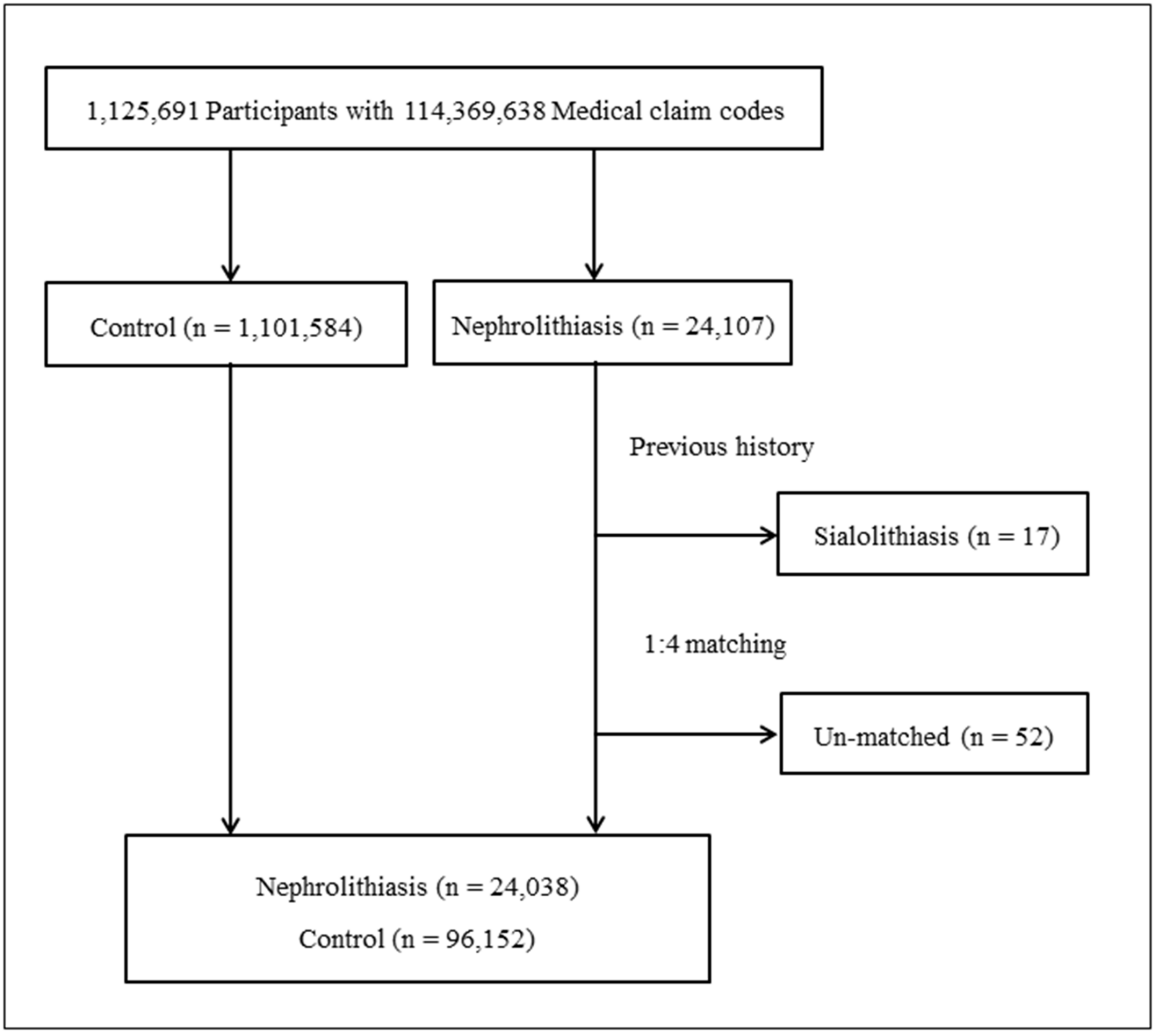
A schematic illustration of the participant selection process that was used in the present study. Out of a total of 1,125,691 participants, 24,038 nephrolithiasis participants were matched with 96,152 control participants for age, group, sex, income group, region of residence, and past medical histories.

The nephrolithiasis patients were matched 1:4 with participants (control group) who were not diagnosed with nephrolithiasis between 2002 and 2013. The control group was selected from the mother population (n = 1,101,584). Matching was for age, group, sex, income group, region of residence, and past medical histories (hypertension, diabetes, and dyslipidemia). To prevent selection bias when selecting the matched participants, the control members were sorted from first to last using a random number order. It was assumed that the control participants were involved at the same time as the matched nephrolithiasis participants (index date). We excluded control patients who died before the index date and participants in both groups with a history of sialolithiasis before the index date. Seventeen participants were excluded from the nephrolithiasis group together with an additional 52 participants for whom we could not find adequate matches. Finally, 24,038 nephrolithiasis patients were matched with 96,152 controls (Fig 1). These individuals were not matched for ischemic heart disease, cerebral stroke, depression, or chronic obstructive pulmonary disorder histories because such strict matching increases the drop-out of the participant due to lack of controls.

### Confounding variables

Age was divided into 5-year intervals: 0–4, 5–9, 10–14…, and 85+ years old. A total of 18 age groups were defined (S1 Table). Income was initially divided into 41 classes (one health aid class, 20 self-employment health insurance classes, and 20 employment health insurance classes) and these were re-categorized into 11 classes (class 1 [lowest income]-11 [highest income]). Region of residence was divided into 16 areas according to administrative district, and regrouped into urban (Seoul, Busan, Daegu, Incheon, Gwangju, Daejeon, and Ulsan) and rural (Gyeonggi, Gangwon, Chungcheongbuk, Chungcheongnam, Jeollabuk, Jeollanam, Gyeongsangbuk, Gyeongsangnam, and Jeju) areas.

The medical histories of participants were evaluated using ICD-10 codes. For accuracy of diagnosis, hypertension (I10 and I15), diabetes (E10-E49), and dyslipidemia (E78) were included if the participants were treated ≥ 2 times, and ischemic heart disease (I24 and I25) and cerebral stroke (I60-I66) if the participants were treated at least once. Depression was defined as F31 (bipolar affective disorder) through F39 (unspecified mood disorder) and individuals were included if they were treated ≥ 2 times by a psychiatrist. Chronic obstructive pulmonary disease (COPD) was defined as previously [24].

### Statistical analysis

The chi-square test was used to compare rates of general characteristics in the nephrolithiasis and control groups. To analyze the hazard ratio of nephrolithiasis to sialolithiasis, a Cox-proportional hazard model was used. In this analysis, crude (simple) and adjusted models (age, sex, income, region of residence, hypertension, diabetes, dyslipidemia, ischemic heart disease, cerebral stroke, depression and COPD histories) were used. For the subgroup analysis, we grouped the participants by age (<30 years old; 30–59 years old; ≥ 60 years old) and sex (male; female). Two-tailed analyses were conducted, and P-values less than 0.05 were considered to indicate significance. The results were analyzed using SPSS v. 21.0 (IBM, Armonk, NY, USA).

## Results

The distribution of age groups (3 subgroups of age), sex, level of income (categorized into 11 levels), region of residence (urban vs. rural), and incidences of hypertension, diabetes, and dyslipidemia were identical in the nephrolithiasis and control groups, since they were matched for these characteristics (Table 1). Higher rates of histories of ischemic heart disease, cerebral stroke, depression, and COPD were observed in the nephrolithiasis group (all P values < 0.05). The overall incidence of sialolithiasis among the nephrolithiasis patients was 0.08% (19/24,038) and 0.1% in the control group (92/96,152, P = 0.447).

**Table 1:** General characteristics of participants.

Characteristics	Total participants		
	Nephrolithiasis (n, %)	Control group (n, %)	P-value
Age (years old)			1.000
< 30	5,464 (22.7)	21,856(22.7)	
≥ 30 and < 60	15,776 (65.6)	63,104 (65.6)	
≥ 60	2,798 (11.6)	11,192 (11.6)	
Sex			1.000
Male	15,509 (64.5)	62,036 (64.5)	
Female	8,529 (35.5)	34,116 (35.5)	
Income			1.000
1 (lowest)	293 (1.2)	1,172 (1.2)	
2	1,350 (5.6)	5,400 (5.6)	
3	1,490 (6.2)	5,960 (6.2)	
4	1,639 (6.8)	6,556 (6.8)	
5	1,988 (8.3)	7,952 (8.3)	
6	2,177 (9.1)	8,708 (9.1)	
7	2,414 (10.0)	9,656 (10.0)	
8	2,733 (11.4)	10,932 (11.4)	
9	3,029 (12.6)	12,116 (12.6)	
10	3,285 (13.7)	13,140 (13.7)	
11 (highest)	3,640 (15.1)	14,560 (15.1)	
Region of residence			1.000
Urban	11,679 (48.6)	46,716 (48.6)	
Rural	12,359 (51.4)	49,436 (51.4)	
Hypertension			1.000
Yes	8,586 (35.7)	34,344 (35.7)	
No	15,452 (64.3)	61,808 (64.3)	
Diabetes Mellitus			1.000
Yes	4,634 (19.3)	18,536 (19.3)	
No	19,404 (80.7)	77,616 (80.7)	
Dyslipidemia			1.000
Yes	7,015 (29.2)	28,060 (29.2)	
No	17,023 (70.8)	68,092 (70.8)	
Ischemic heart disease			<0.001*
Yes	1,514 (6.3)	5,084 (5.3)	
No	22,524 (93.7)	91,068 (94.7)	
Stroke			0.002*
Yes	2,077 (8.6)	7,721 (8.0)	
No	21,961 (91.4)	88,431 (92.0)	
Depression			<0.001*
Yes	2,117 (8.8)	7,244 (7.5)	
No	21,921 (91.2)	88,908 (92.5)	
COPD			<0.001*
Yes	1,633 (6.8)	5,458 (5.7)	
No	22,405 (93.2)	90,694 (94.3)	
Sialolithiasis			0.447
Yes	19 (0.1)	92 (0.1)	
No	24,019 (99.9)	96,060 (99.9)	

COPD: chronic obstructive pulmonary disease

* Chi-square test, Significance at P < 0.05

Crude and adjusted hazard ratios (HR) of nephrolithiasis for sialolithiasis were not statistically significant. The adjusted HR of nephrolithiasis for sialolithiasis was 0.81 (95% confidence interval [CI] = 0.49–1.33, P = 0.399, Table 2). In the subgroup analysis according to age (<30, 30–59, ≥ 60 years old), nephrolithiasis did not significantly elevate the risk of sialolithiasis (Table 3). In the male and female subgroups, crude and adjusted HRs did not differ between the nephrolithiasis and control groups.

**Table 2:** Crude and adjusted hazard ratios (95% confidence interval) of nephrolithiasis for sialolithiasis.

Characteristics	Sialolithiasis			
	Crude	P-value*	Adjusted†	P-value*
Nephrolithiasis	0.82 (0.50–1.35)	0.448	0.81 (0.49–1.33)	0.399
Control	1.00		1.00	

* Cox-proportional hazard regression model

^†^ Adjusted model for age, sex, income, region of residence, hypertension, diabetes, dyslipidemia, ischemic heart disease, cerebral stroke, depression and chronic obstructive pulmonary histories.

**Table 3:** Subgroup analysis of crude and adjusted hazard ratios (95% confidence interval) of nephrolithiasis for sialolithiasis according to age and sex.

Characteristics	Sialolithiasis			
	Crude	P-value*	Adjusted†	P-value*
Age < 30 years old (n = 27,320)				
Nephrolithiasis	0.41 (0.12–1.36)	0.145	0.41 (0.13–1.34)	0.141
Control	1.00		1.00	
Age ≥ 30 and < 60 years old (n = 78,880)				
Nephrolithiasis	0.86 (0.46–1.60)	0.627	0.82 (0.44–1.53)	0.535
Control	1.00		1.00	
Age ≥ 60 years old (n = 13,990)				
Nephrolithiasis	2.29 (0.67–7.81)	0.187	2.48 (0.72–8.48)	0.149
Control	1.00		1.00	
Male (n = 77,545)				
Nephrolithiasis	0.81 (0.43–1.56)	0.535	0.80 (0.42–1.54)	0.508
Control	1.00		1.00	
Female (n = 42,645)				
Nephrolithiasis	0.84 (0.39–1.80)	0.658	0.81 (0.38–1.74)	0.594
Control	1.00		1.00	

* Cox-proportional hazard regression model

^†^ Adjusted model for age, sex, income, region of residence, hypertension, diabetes, dyslipidemia, ischemic heart disease, cerebral stroke, depression and chronic obstructive pulmonary histories.

## Discussion

This study found no evidence that the occurrence of nephrolithiasis is associated with an increased risk of sialolithiasis, after adjusting for age, sex, level of income, region of residence, hypertension, diabetes, and dyslipidemia. In addition, the incidence of sialolithiasis was not affected by nephrolithiasis in subgroups for age and sex. These finding provide evidence that the causes of sialolithiasis and nephrolithiasis may differ.

When we searched PubMed and Scopus using the terms [sialolithiasis], [nephrolithiasis], [stone], [calculus], and [calculi] for papers published in English from January 1985 to June 2017, we encountered three studies that evaluated the association between nephrolithiasis and sialolithiasis. Two were based on a small number of patients in a hospital database [17,19]; one, a retrospective study of 245 patients with sialolithiasis treated for 20 years reported that patients with salivary stones were more likely to develop nephrolithiasis [17]. The other, a retrospective cohort study which involving 153 patients with sialolithiasis reported that the occurrence of nephrolithiasis or cholelithiasis was not associated with sialolithiasis, in agreement with our findings [19]. These two studies evaluated the incidence of nephrolithiasis among individuals with sialolithiasis, whereas we evaluated the rates of sialolithiasis among matched nephrolithiasis and control individuals. To date, only one other study has evaluated the association between sialolithiasis and nephrolithiasis using a large public population database. Wu et al. used the Taiwan Longitudinal Health Insurance Database to evaluate 966 patients with sialolithiasis and 2,898 age- and sex-matched controls [18]. The odds ratio for prior nephrolithiasis in patients with sialolithiasis relative to the controls was 4.74 (95% CI = 3.41–6.58, P < 0.001) in that study. The difference between the results of our study and those of Wu et al. may be due to the different ethnic groups examined. Indeed, the incidence rates and prevalence of renal stone diseases differ between ethnic groups [25]. In addition, our procedure for selecting sialolithiasis and nephrolithiasis patients was more accurate, because we only selected patients who were seen at least twice for the relevant disorder. The absence of an association is likely to have greater statistical significance when the study population is large, as in our case.

Many reports support our evidence that the main causes of nephrolithiasis and sialolithiasis are different. The cause of nephrolithiasis depends on whether the stone is a calcium stone or a non-calcium stone. Calcium renal stones are caused by hypercalciuria, hyperuricosuria, hyperoxaluria, hypocitraturia, abnormal urine pH, and low urine volume [26]. Hypercalciuria was present in up to 60% of the nephrolithiasis cases in the literature [27]. Non-calcium renal stones can be caused by uric acid, cystinuria, and, rarely, by infection [1]. Genetic polymorphisms have been suggested to be responsible for both calcium and non-calcium renal stones [28, 29].

Various causes of sialolithiasis have been proposed, including tobacco smoking [19], chronic infection of the oral cavity and periodontitis [30], salivary duct trauma or stenosis, reduced fluid intake, and medication including diuretics leading to stasis and decreased flow of saliva [31]. Unlike nephrolithiasis, sialolithiasis is considered to be caused by local factors, such as causes arising from the salivary ductal system, stasis or content of the saliva, and adjacent oral cavity diseases, as opposed to systemic diseases or medication. Many studies have shown that decreased salivary flow and chronic obstructive sialadenitis are the main causes of sialolith formation [32,33]. One interesting study examined the presence of bacterial genes in salivary stones by polymerase chain reaction, and identified oral bacteria, mostly *Streptococcus* species, in all of them [34]. Another study examined salivary stones by transmission and scanning electron microscopy, and again detected the accumulation of microorganisms inside the calculi [35]. Similarly, a case-control study of 987 cases of sialolithiasis found that prior chronic periodontitis was an independent risk factor for the development of sialolithiasis, with an odds ratio of 1.37 (95% CI, 1.19–1.56) [30]. Another study suggested an association between dental calculi and salivary calculi and suggested that oral bacteria were a common cause [34].

Unlike in nephrolithiasis, the literature points to no direct correlation between systemic factors such as calcium metabolism and sialolithiasis [1,36]. This finding is supported by the fact that the majority of salivary stones involve one salivary gland, and simultaneous sialolithiasis in multiple salivary glands is very rare [37,38]. Paterson et al. have pointed out that although patients with hyperparathyroidism have high concentrations of calcium and phosphate in both urine and saliva, nephrolithiasis is common in that condition but sialolithiasis is relatively rare [39]. The authors emphasize that abnormal bone biochemistry need not be investigated routinely in sialolithiasis, unlike in nephrolithiasis. Specific proteins including proline and statherin play an important role in the formation of sialolithiasis, but not in nephrolithiasis [40]. Sherman et al. found no association between water hardness and sialolithiasis, and suggested that high calcium intake is irrelevant to the development of sialolithiasis [41]. However, a recent epidemiological study suggested a close relationship between the drinking water concentration of calcium/magnesium and the incidence of sialolithiasis [42].

The strength of the present study is the large number of participants with nephrolithiasis (n = 24,038) and hence its high statistical power. The study represents the whole population of South Korea, because every citizen has to join the public medical insurance scheme. This is the second study to evaluate the association between sialolithiasis and nephrolithiasis using a public population database. In our study, the participants were followed for a long period, with a maximum of 12 years. Another advantage of this study is that comprehensive medical records were available for every participant, which enabled us to adjust for confounding factors.

This is the first study using a public national database to report a lack of association between the incidence of nephrolithiasis and sialolithiasis. However it has several limitations. First, it was not a randomized clinical trial. However, we matched each nephrolithiasis patient with controls adjusting for age, sex, region of residence, medical disease including hypertension, diabetes, and dyslipidemia. Also, it is difficult to perform a randomized clinical study with a long follow-up period. Second, data on dietary habits, climate, and body-mass index were not available. Third, the participants were all Koreans, and it is well known that stone disease differs between ethnic groups [4]. Confirmatory studies involving different ethnic groups are required.

## Conclusion

We found no evidence that nephrolithiasis is associated with an increased risk of sialolithiasis. We suggest that whereas nephrolithiasis is accompanied by high concentrations of calcium, uric acid, citrate, and oxalate in the urine, sialolithiasis is a multifactorial disease associated with chronic sialadenitis, salivary contents, and the anatomy of the salivary duct system. We propose that routine evaluation for sialolithiasis in all patients with nephrolithiasis is not necessary.

## Supporting information

S1 Table Age information of participants. (DOCX)
