# Supplementary material for: Lack of evidence that nephrolithiasis increases the risk of sialolithiasis: A longitudinal follow-up study using a national sample cohort
Source: PLoS One. 2018 Apr 26;13(4):e0196659. doi: 10.1371/journal.pone.0196659 (PMC5919636; doi:10.1371/journal.pone.0196659)
Supplement: S1 Table — (DOCX) [file pone.0196659.s001.docx]

**S1 Table** Age Information of Participants

| Variable | | Total participants | | |
| --- | --- | --- | --- | --- |
|  |  | Nephrolithiasis (n, %) | Control group (n, %) | P-value* |
| Age (years old) | |  |  | 1.000 |
|  | 0-4 | 41 (0.2) | 164 (0.2) |  |
|  | 5-9 | 206 (0.9) | 824 (0.9) |  |
|  | 10-14 | 425 (1.8) | 1,700 (1.8) |  |
|  | 15-19 | 901 (3.7) | 3,604 (3.7) |  |
|  | 20-24 | 1,652 (6.9) | 6,608 (6.9) |  |
|  | 25-29 | 2,239 (9.3) | 8,956 (9.3) |  |
|  | 30-34 | 2,783 (11.6) | 11,132 (11.6) |  |
|  | 35-39 | 2,845 (11.8) | 11,380 (11.8) |  |
|  | 40-44 | 3,426 (14.3) | 13,704 (14.3) |  |
|  | 45-49 | 2,869 (11.9) | 11,476 (11.9) |  |
|  | 50-54 | 2,141 (8.9) | 8,564 (8.9) |  |
|  | 55-59 | 1,712 (7.1) | 6,848 (7.1) |  |
|  | 60-64 | 1,478 (6.1) | 5,912 (6.1) |  |
|  | 65-69 | 779 (3.2) | 3,116 (3.2) |  |
|  | 70-74 | 359 (1.5) | 1,436 (1.5) |  |
|  | 75-79 | 146 (0.6) | 584 (0.6) |  |
|  | 80-84 | 31 (0.1) | 120 (0.1) |  |
|  | 85+ | 6 (0.0) | 24 (0.0) |  |

*Chi-square test
